# Dysbiosis of Gastric Mucosal Fungal Microbiota in the Gastric Cancer Microenvironment

**DOI:** 10.1155/2022/6011632

**Published:** 2022-03-16

**Authors:** Zhenzhan Zhang, Hao Feng, Yaopeng Qiu, Zhou Xu, Qingfeng Xie, Wenfu Ding, Hao Liu, Guoxin Li

**Affiliations:** Department of General Surgery & Guangdong Provincial Key Laboratory of Precision Medicine for Gastrointestinal Tumor, Nanfang Hospital, Southern Medical University, Guangzhou, China

## Abstract

**Background:**

Microbes have been shown to contribute to gastric cancer (GC), gastric bacteria and viruses are associated with gastric carcinogenesis. However, the relationship between gastric fungi and GC is still unclear. Our aim was to evaluate the gastric fungal microbiota in the GC microenvironment.

**Methods:**

Gastric fungal microbiome profiling was performed with internal transcribed spacer (ITS) rDNA sequencing in primary tumor and corresponding paired normal mucosal tissues from 61 GC patients. Differences in microbial composition, taxa diversity, and predicted function were further analyzed.

**Results:**

Dysbiosis of gastric mucosal fungal microbiome was observed between the tumor and normal groups in GC. The tumor group had a higher abundance of certain taxa than the normal group. In the taxa classification, the abundances of *Pezizomycetes*, *Sordariales*, *Chaetomiaceae*, and *Rozellomycota* were lower in the tumor group than in the normal group. At the genus level, *Solicoccozyma* (*P* = 0.033) was found in higher abundance and was differentially enriched in the tumor group with Lefse analysis. Additionally, *Solicoccozyma* accounted for 0.3% of gastric fungi in the GC microenvironment. Twenty-seven of the 61 GC patients showed positive *Solicoccozyma* expression in tumors. *Solicoccozyma*-positive expression in tumors was associated with the Bormann classification and nerve invasion. *Solicoccozyma* was considered a gastric fungal marker to classify stage I and stage II-IV GC patients with an area under the receiver-operating curve (AUC) of 0.7061, as well as to classify the nerve invasive and nonnerve invasive tumors from GC patients with an AUC of 0.6978. Functional prediction indicated that the positive expression of *Solicoccozyma* in tumors was associated with the amino acid- and carbohydrate-related metabolic pathways in GC.

**Conclusions:**

This study revealed a novel perspective on the role of *Solicoccozyma* in tumors and a theoretical basis for therapeutic targets against GC.

## 1. Introduction

Gastric cancer (GC) is considered a common gastrointestinal malignancy and the leading cause of human cancer-related mortality worldwide [1]. Although the incidence and mortality rates are declining with therapeutic advances, GC is a global medical burden [2]. Therefore, it is urgent to understand the mechanisms of gastric tumorigenesis and find therapeutic targets against GC.

Microbes are considered a key component of the tumor microenvironment. The human stomach harbors a great number of microbes including bacteria, fungi, and viruses, and *Helicobacter pylori* and Epstein-Barr virus as important risk factors have been shown to contribute to gastric tumorigenesis [3, 4]. Other microbes (such as fungi) may have unknown potential effects on GC. Over the past decade, dysbiosis of the fungal microbiota has been reported to be associated with tumorigenesis in the pancreas, colon, prostate, breast, and stomach. Pathogenic fungal mycobiomes (such as *Malassezia*) drive the complement cascade to promote pancreatic oncogenesis by activating MBL [5]. The oral fungal microbiotas were associated with colorectal carcinogenesis [6]. However, gut-derived fungi mediated inflammasome activation via the SYK-CARD9 signaling axis to restrict colitis and colon cancer progression [7]. Moreover, *Candida albicans* triggered glycolysis in tumor-associated macrophages, which induced IL-22 secretion from innate lymphoid cell to promote colon cancer [8]. A fungal microbiota signature was observed in prostate cancer tissues compared with benign prostate hyperplasia controls [9]. The fungal signatures of the four major breast cancer subtypes were obtained by using pathochip technology [10]. In the human stomach, fungal colonization of the gastric mucosa influenced on the course of gastric ulcer healing and the “fungal” gastric ulcers tend to be larger in diameter and are more often suspected to be malignant than “nonfungal” gastric ulcers [11, 12]. Gastric fungal microbiomes may lead to malignant process. Gastric mycobiota imbalance has been associated with gastric carcinogenesis, and *Candida albicans* has been used as a fungal biomarker to distinguish GC from the control [13]. However, the potential association between the gastric fungal microbiota and GC is still unclear. The role of gastric fungal microbiota in the GC microenvironment needs to be further explored.

In this study, we analyzed the gastric fungal composition in primary tumor and corresponding paired normal mucosal tissues from 61 GC patients by ITS rDNA sequencing. We demonstrated the dysbiosis of gastric fungal microbiota in the GC microenvironment, further revealing the role of *Solicoccozyma* in GC and providing a theoretical basis for the antifungal prevention and therapeutic targets against GC.

## 2. Methods

### 2.1. Patients and Sample Collection

A total of 61 patients with GC were enrolled from March 2020 to September 2020 from the Nanfang Hospital, Southern Medical University (Guangdong, China) in this study. All patients provided informed consent for obtaining specimens, and the study was approved by the Clinical Research Ethics Committees of the Nanfang Hospital, Southern Medical University. None of the GC patients had used antibiotics within 2 months or received any preoperative chemotherapy, radiotherapy, and immunotherapy. The patient characteristics are shown in Table 1. These tissue samples were obtained from GC patients receiving surgical gastrectomy. All tissue samples were excised after gastrectomy, collected and stored in sterile collectors, and frozen immediately at -80°C. Finally, a total of 122 gastric tissue samples including the primary tumor and corresponding paired normal tissues from 61 GC patients were collected for fungal microbiota analysis.

### 2.2. DNA Extraction, PCR Amplification, and Its rDNA Gene Sequencing

The tissue samples were disrupted and digested for DNA extraction. The quality of DNA was detected by 1.2% agarose gel electrophoresis. PCR was performed to amplify target fragments with fungal-universal primers according to the manufacturer's protocols. The optimal sequence length of the target fragment was 280-450 bp. Fungal primer sequences were designed and reported in previous studies [14, 15]. The universal primer sequences used were ITS5-1737F: 5′-GGAAGTAAA AGTCGTAACAAGG-3′ and ITS2-2043R: 5′-GCTGCGTTCTTCATCGATGC-3′. The primers amplified the ITS1-5F region. Then, magnetic beads were pacificated and recovered, and the amplified products were sent for fluorescence quantification and to further prepare a sequencing library by using a Nano DNA LT Library Prep Kit (Illumina) and Agilent High Sensitivity DNA Kit. After quantification of the library by a Quant-iT Pico Green dsDNA Assay Kit at 2 nM concentrations, high-throughput sequencing was performed on the NovaSeqPE250 platform following standard platform protocols. For quality control of sequencing data, the DADA2 method was used for sequence denoising or clustering by QIIME2 software, and R script software was used to determine sequence length distribution statistics [16]. The species taxonomy analysis based on the fungal ITS rDNA sequences was performed by using the UNITE database (Release 8.0), and the relative abundance of the amplicon sequence variants (ASVs) or operational taxonomic units (OTUs) were obtained [17].

### 2.3. Microbiota Taxonomic Composition Analysis and Functional Prediction

The relative abundance of ASVs or OTUs were described to analyze the alpha- and beta-diversity of the fungal microbiota. Alpha-diversity indexes of microbiota included the Chao1, Observed_species, Pielou_e, Shannon, Simpson, Faith's_pd, and Good's_coverage index. In beta-diversity analysis, principal coordinates analysis (PCoA) and nonmetric multidimensional scaling (NMDS) were used to analyze the grouping differences in samples. Rarefaction curves displayed the number of ASVs/OTUs in different samples or groups under the same sequencing depth to measure the diversity of each sample or group [18]. A Venn diagram and heatmap were used to indicate the distribution of the gastric mucosal fungal microbiota based on the average abundance of ASV/OTUs. In addition, with the linear discriminant analysis (LDA) effect size (LEfSe) analysis, differences with LDA scores greater than 2 and *P* value < 0.05 were considered significant [19]. Principal component analysis (PCA) and orthogonal partial least squares discriminant analysis (OPLS-DA) were used to indicate microbial composition differences between samples. To predict the functions of the microbiota based on the abundance and occurrence of the genome sequences of specific marker genes [20, 21], phylogenetic investigation of communities by reconstruction of unobserved states (PICRUSt, version.2.0) software was used, and the enrichment of metabolic pathways based on the MetaCyc database was evaluated to predict functional capabilities and metabolic processes.

### 2.4. Statistical Analysis

The Mann–Whitney *U* test was performed for the continuous variables, and Pearson's chi-square test was used for the categorical variables. Statistical analysis was performed using SPSS V22.0 (SPSS Inc. Chicago, IL). GraphPad Prism version 8.0 (San Diego, CA) was used for the preparation of graphs. All tests of significance were two-sided, and *P* value < 0.05 was considered statistically significant.

## 3. Results

### 3.1. Dysbiosis of Gastric Mucosal Fungal Microbiota in the GC Microenvironment

**Figure 1:**
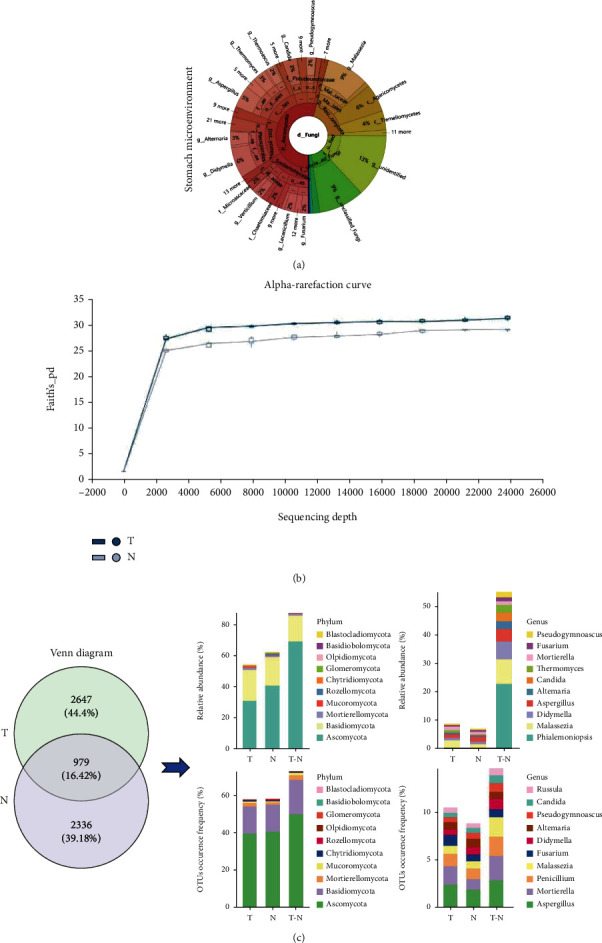
Alteration of gastric mucosal fungal microbiota between the tumor and normal group. (a) Krona pie charts were established to indicate the distribution of gastric mucosal fungal microbiomes in the stomach microenvironment; (b) alpha rarefaction curves were used to estimate the species richness of the fungal microbiota between the two groups; (c) the Venn diagram illustrated the overlapping gastric mucosal fungal microbiota between the two groups, and the distribution and abundance of overlapping fungi was evaluated based on the sequence information of the OTUs at the phylum and genus level.

We collected primary tumor and corresponding paired normal mucosal tissues from 61 GC patients and divided them into two groups according to the tumor (T) and normal (N) group. These gastric tissues were sent for ITS rDNA sequencing for gastric fungal microbiome profiling. The distribution of gastric fungi in the stomach microenvironment was shown in a Krona pie chart (Figure 1(a)). Additionally, the alpha-rarefaction curves of fungal OTUs showed a higher richness in the tumor group than in the normal group (Figure 1(b)). The Venn diagram illustrated the overlapping of fungal OTUs between the two groups and revealed that a higher abundance of unique OTUs was observed in the tumor group. Moreover, based on the sequence information of the species OTUs, the distribution and abundance of the overlapping fungal microbiota was shown at the phylum and genus level (Figure 1(c)). However, the alpha-diversity analysis, including the Chao1, Observed species, Pielou_e, Shannon, Simpson, Faith's_pd, and Good's_coverage index showed negative differences in species richness between the tumor and normal group (Supplementary Figure 1). The beta-diversity analysis, including PCoA and NMDS, suggested that the distribution of the fungal community between the two groups could not be aggregated separately due to interindividual variation in GC patients (Supplementary Figure 2). Taken together, these results indicated the dysbiosis of gastric mucosal fungal microbiota in the GC microenvironment.

### 3.2. Differential Microbial Taxa in the GC Microenvironment

**Figure 2:**
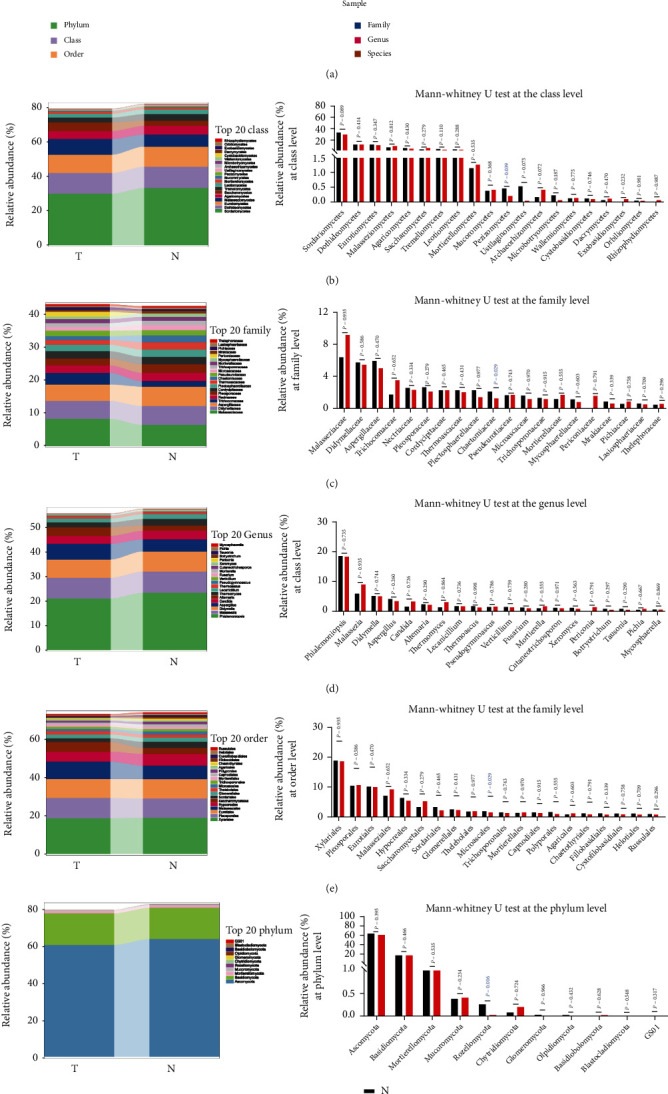
Differential taxa composition between the tumor and normal group in GC. (a) The fungal composition in primary tumor and corresponding paired normal mucosal tissues from 61 GC patients was displayed at different classification levels, including the phylum, class, order, family, genus, and species levels; (b–f) the abundance and distribution of the top 20 fungal taxa were described between the two groups at the class, family, genus, order, and phylum levels and were analyzed by the Mann–Whitney *U* test.

To further identify the taxa composition of fungal microbiotas between the tumor and normal groups, the number of microbial taxa in primary tumor and corresponding paired normal mucosal tissues from 61 GC patients was evaluated at the different taxa classification levels (Figure 2(a)). We described the abundance of the top 20 fungal taxa between the two groups and found that a significantly lower abundance of *Pezizomycetes* (*P* = 0.039) at the class level, *Chaetomiaceae* (*P* = 0.029) at the family level, *Sordariales* (*P* = 0.021) at the order level, and *Rozellomycota* (*P* = 0.016) at the phylum level in the tumor group than in the normal group based on the Mann–Whitney *U* test (Figures 2(b)–2(f)). These results suggested that these differential fungal taxa may be important factors that trigger the imbalance of gastric mucosal fungal microbiota in the GC microenvironment.

### 3.3. The Role of *Solicoccozyma* in the GC Microenvironment

**Figure 3:**
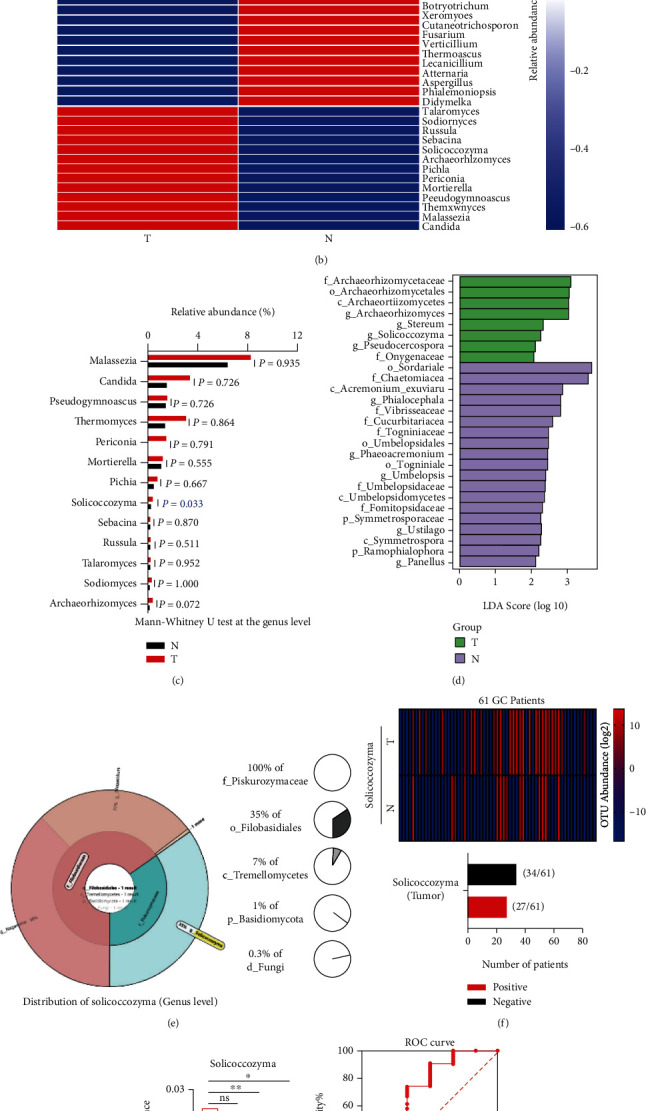
Fungal microbiome profiling revealed the role of *Solicoccozyma* in the GC microenvironment. (a) A heatmap described the average abundance of the top 50 gastric fungi at the genus level; (b) a cluster heatmap indicated the differentially enriched gastric fungal microbiomes between the two groups; (c) the 13 fungal microbiotas that had higher abundances and were differentially enriched in the tumor group by comparison to the normal group based on the Mann–Whitney *U* test; (d) LEfSe identified the taxa with the greatest differences in abundance between the tumor and normal group, the taxa with a significant LDA threshold value of >2 and *P* value < 0.05 are shown (p: phylum, c: class, o: order, f: family, g: genus, s: species); (e) the distribution of *Solicoccozyma* account for a percentage of gastric fungi in the GC microenvironment; (f) a cluster heatmap showed the abundance of *Solicoccozyma* in the primary tumor and corresponding paired normal tissues from 61 GC patients, and GC patients with positive *Solicoccozyma* expression in tumor was described; (g) the abundance of *Solicoccozyma* in GC patients with stages I, II, III, and IV; (h) the ROC curves of *Solicoccozyma* provided an area under the receiver-operating characteristic curve (AUC) for the classification of the stage I and stage II-IV GC patients; (i) the abundance of *Solicoccozyma* in GC patients with nerve invasion and nonnerve invasion; (j) the AUC value of *Solicoccozyma* for the classification of the nerve invasive and nonnerve invasive GC patients.

A heatmap described the average abundance of the top 50 fungal microbiotas at the genus level in the tumor and normal groups (Figure 3(a)). Furthermore, a cluster heatmap displayed the differentially enriched gastric fungal microbiotas between the two groups (Figure 3(b)). PCA and OPLS-DA were applied to classify the main and separated components of the gastric mucosal fungal microbiota between the two groups. The separated components of fungal microbiotas in the two groups were excluded (Supplementary Figure 3). At the genus level, only *Solicoccozyma* was found to have a significantly higher abundance in the tumor group than in the normal group (*P* = 0.033) with the Mann–Whitney *U* test (Figure 3(c)), while the other fungal microbiotas that were differentially enriched in the normal group showed no differences between the two groups (Supplementary Figure 4). In addition, discriminant analysis using LEfSe showed that 27 fungi were significantly different between the two groups and that *Solicoccozyma* was differentially enriched in the tumor group (Figure 3(d)). The random forest classifier at the genus level also showed the importance of *Solicoccozyma* in the grouping difference (Supplementary Figure 5A). A Krona pie chart showed that the distribution of *Solicoccozyma* accounted for 0.3% of gastric fungi in the GC microenvironment (Figure 3(e)). *Solicoccozyma* attracted our attention for analysis. A total of 27 of 61 GC patients with positive *Solicoccozyma* expression were included (Figure 3(f)). We found that positive *Solicoccozyma* expression in tumors was associated with the Bormann classification (*P* = 0.019) and the nerve invasion (*P* = 0.044) in GC patients (Table 2). Surprisingly, we observed a significantly higher abundance of *Solicoccozyma* in GC patients with stage I or nonnerve invasion than in GC patients with stage II-IV or nerve invasion (Figures 3(g) and 3(i)). Subsequently, we evaluated the accuracy based on the ROC curve with an area under the AUC value of 0.7061 to classify stage I and stage II-IV GC patients (Figure 3(h)) and the AUC value of 0.6978 to classify the nerve invasive and nonnerve invasive GC patients (Figure 3(j)). Thus, *Solicoccozyma* has the potential to be a gastric fungal marker to classify GC patients with stage I and stage II-IV as well as classify GC patients with nonnerve invasion and nerve invasion.

*Solicoccozyma aeria* is one of the main species of *Solicoccozyma*. At the species level, we found that the OTU abundance of *Solicoccozyma aeria* was significantly elevated in the tumor group (*P* = 0.043) (Supplementary Figure 5B). Finally, 21 of 61 GC patients had positive *Solicoccozyma aeria* expression in tumors (Supplementary Figure 5C). Positive *Solicoccozyma aeria* expression was also associated with the Bormann classification (*P* = 0.029), nerve invasion (*P* = 0.016), and lymphatic vessel invasion (*P* = 0.026) in GC patients (Supplementary Table 1). Based on the AUC values, similar to *Solicoccozyma*, *Solicoccozyma aeria* could be a gastric fungal species marker to classify GC patients with stage I and stage II-IV and to classify GC patients with nonnerve invasion and nerve invasion (Supplementary Figures 5D–5G). Overall, the results suggested that *Solicoccozyma* plays a key role in the GC microenvironment.

### 3.4. Functional Prediction of Fungal Microbiota in the GC Microenvironment

**Figure 4:**
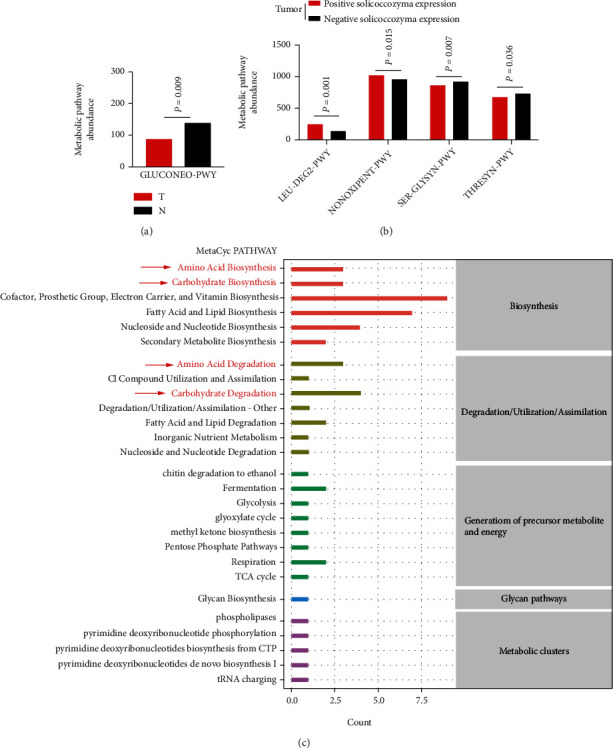
Functional prediction of the gastric mucosal fungal microbiota. (a) Metabolic pathways were enriched between the tumor and normal group; (b) the metabolic pathways were enriched in GC patients with *Solicoccozyma-*positive expression in tumors; (c) based on the OTU occurrence of the fungal microbiota and MetaCyc database, the metabolic processes were enriched and shown (red markers indicated amino acid- and carbohydrate-related metabolic processes).

The functional capacity of the microbiota was predicted by using the PICRUSt.2 and MetaCyc databases. Based on the pathway abundance, we found that the gastric fungi in the tumor group were predicted to be associated with GLUCONEO-PWY (*P* = 0.009) (Figure 4(a)). GLUCONEO-PWY is an amino acid- and carbohydrate-related pathway. Moreover, *Solicoccozyma*-positive expression in tumors was also predicted to be associated with amino acid- and carbohydrate-related pathways, such as LEU-DEG2-PWY (*P* = 0.0001), NONOXIPENT-PWY (*P* = 0.015), SER-GLYSYN-PWY (*P* = 0.007), and THRESYN-PWY (*P* = 0.036) (Figure 4(b)). In addition, the functional prediction of *Solicoccozyma aeria* was similar to that of *Solicoccozyma* expression in tumors (Supplementary Figure 5H). Based on the OTU occurrence of the fungal microbiota, the processes of biosynthesis, degradation/utilization/assimilation, generation of precursor metabolite and energy, glycan pathways, and metabolic clusters were further analyzed. We observed that amino acid- and carbohydrate-related metabolic processes were enriched (Figure 4(c)). The results indicated that *Solicoccozyma* may affect amino acid- and carbohydrate-related metabolic processes in the GC microenvironment.

## 4. Discussion

The human stomach is surrounded by the multiple microbiotas, including bacterial, fungal, and other communities. In recent years, the association between the microbiota and gastrointestinal tumor has been recognized gradually. The imbalance of gastric microbiota plays key roles in promoting tumor initiation, progression, and distant metastasis in GC [3, 4]. Although the human gut microbiota was involved in cancers of the gastrointestinal tract, and the alteration of the gut microbiome was a predictive marker for GC patients [22, 23], the human gastric microbiota has great advantages in displaying dynamic alterations and direct interactions with GC in the same human stomach microenvironment.

In this study, we performed ITS rDNA sequencing with the NovaSeqPE250 platform to analyze the composition of gastric fungi between primary tumor tissues and corresponding paired normal tissues in GC patients. The NovaSeqPE250 platform was used to sequence the fungal ITS1 region amplicon, which was 280-450 bp long for certain common fungal species. However, other fungal species with the same primer pair have an ITS1 region amplicon over 500 bp long, which is difficult to amplify under the NovaSeqPE250 platform. Our study was limited and analyzed common fungal species with the 280-450 bp-long ITS1 region amplicons in the GC microenvironment. We observed dysbiosis of gastric mucosal fungal microbiome between the tumor and normal group in GC patients. At the genus level, *Solicoccozyma* was significantly elevated in the tumor group. We found that *Solicoccozyma* not only appeared and was significantly enriched in the tumor group but also appeared in the normal group. *Solicoccozyma* may not be a tumor-specific gastric fungi in the GC microenvironment. However, in gastric tumor tissues, *Solicoccozyma* could be considered a gastric fungal marker to classify stage I and stage II-IV GC patients, as well as to classify nerve invasive and nonnerve invasive GC patients. A higher abundance of *Solicoccozyma* was present in GC patients with stage I or nonnerve invasion than in GC patients with stage II-IV or nerve invasion. Moreover, the analysis of clinical characteristics also provided evidence that GC patients with positive *Solicoccozyma* expression in tumors have less tumor invasion and nerve invasion. Therefore, *Solicoccozyma* may play a protective role in suppressing the development of GC.

Here, we first report the important role of *Solicoccozyma* in GC. Few other studies have reported the role of *Solicoccozyma*. *Solicoccozyma* is an environmental fungus. *Solicoccozyma* is a yeast strain and has three species, *Solicoccozyma aeria*, *Solicoccozyma terricola*, and *Solicoccozyma terrea* [24, 25]. *Solicoccozyma* has been shown to adapt to a wide range of acidic soil microenvironment, such as those with pH ranges of 4.0-4.7 and 5.4-5.6 [26]. Notably, *Solicoccozyma* may have antiacid microbial characteristics. Because the pH of the acidic soil environment is similar to the pH of the acidic human stomach environment in vivo, the possibility of *Solicoccozyma* survival in the human GC microenvironment could be conditional upon gastric acid secretion. Moreover, *Solicoccozyma* was found to be present at high relative abundance in mice, pigs, and humans. In pig model, *Solicoccozyma* derived from the colonic fungal community was related to the response of the composition of dietary carbohydrates (DCHO) and degraded DCHO in the colon [27]. *Solicoccozyma aeria* derived from mouse gut fungal mycobiota was significantly elevated and associated with appetite suppression and IL-17R signaling activation under high temperature and high humidity (HTHH) conditions (32°C ± 2°C, relative humidity 95%). *Solicoccozyma aeria* were isolated and obtained from the feces of a patient under HTHH conditions. In addition, the abundance of *Solicoccozyma aeria* was higher in human saliva under HTHH conditions compared to the controls [28]. Because of the thermal tolerance of fungi, most of the environmental fungal species cannot colonize or even survive at human body temperature, and fungi from mammals such as humans had greater thermal tolerances than isolates from soils and plants. Every 1°C increase in the 30°C-40°C range excluded an additional 6% of fungal isolates [29]. Most of the environmental fungal species do not exist well in the human body. However, *Solicoccozyma aeria* has a high thermal tolerance and survives under high temperature conditions (32°C ± 2°C), similar to the human body temperature. Most importantly, *Solicoccozyma aeria* has been found to exist in human saliva and feces, which also provided evidence that *Solicoccozyma aeria* is able to grow at the human body temperature. It is very important to understand the possibility of *Solicoccozyma* survival in the human body. *Solicoccozyma aeria* has the ability to be a key conditional pathogen enriched in the human digestive tract, such as in the esophagus, stomach, and intestine. Based on the evidence of its antiacid microbial characteristics and thermal tolerance in the human body, *Solicoccozyma* could be further excluded as nonhuman primate host-derived microbial contamination. More experiments, including the gastric tissue-derive fungal culture method and fungal-specific 18S FISH staining, are needed to further validate whether *Solicoccozyma* or *Solicoccozyma aeria* was indeed colonized and expressed on the gastric mucosal tissue surface. Taken together, at the genus level, *Solicoccozyma* expression was associated with GC. However, the microbial functions and mechanisms of *Solicoccozyma* are limited and need to be further proven.

In conclusion, in contrast to previous studies focusing on gastric bacteria and viruses, our study demonstrated the potential relationship of gastric mucosal fungal microbiota dysbiosis and GC and revealed the role of *Solicoccozyma* in the GC microenvironment for the first time. *Solicoccozyma* may serve as a gastric fungal marker for the diagnosis of stage I and nonnerve invasion in GC patients. Targeting *Solicoccozyma* in the GC microenvironment may be a promising therapy against GC.

## Figures and Tables

**Table 1:** Patient characteristics (*n* = 61).

Characteristics	
Age (years) [median (range)]	56 (22-86)
Gender (male/female)	43/18
Body mass index (kg/m^2^) [median (range)]	22.1 (16.6-35.1)
Tumor max size (cm) [median (range)]	4.6 (0.5-15)
Tumor location (upper/middle/lower)	19/12/30
Tumor differentiation (high/moderately/poor)	4/19/38
Lauren type (diffuse/intestinal/mix)	36/17/8
Bormann type (I/II/III/IV)	2/5/42/12
Tumor depth (T1/T2/T3/T4)	7/2/20/32
Lymph node involvement (N0/N1/N2/N3)	17/4/12/28
Distant metastasis (M0/M1)	51/10
Pathological stage (I/II/III/IV)	6/9/34/12
Lymphatic vessel invasive (D2-40) (yes/no)	20/41
Vascular invasive (CD31) (yes/no)	15/46
Nerve invasive (S-100) (yes/no)	46/15

**Table 2:** Clinicopathological characteristics of *Solicoccozyma* at the genus level in GC patients.

Characteristics	*Solicoccozyma* expression		*P* value
	Positive (*n* = 27)	Negative (*n* = 34)	
Age			
≥60	13 (48.1%)	14 (41.2%)	
<60	14 (51.9%)	20 (58.8%)	0.586
Gender			
Male	17 (63.0%)	26 (76.5%)	
Female	10 (37.0%)	8 (23.5%)	0.251
Body mass index			
<18	1 (3.70%)	2 (5.9%)	
18-24	19 (70.4%)	22 (64.7%)	
>24	7 (25.9%)	10 (29.4%)	0.868
Tumor location			
Upper	9 (33.3%)	10 (29.4%)	
Middle/lower	18 (66.7%)	24 (70.6%)	0.743
Tumor differentiation			
High	3 (11.1%)	1 (2.9%)	
Moderately/poor	24 (88.9%)	33 (97.1%)	0.200
Lauren classification			
Diffuse	13 (48.2%)	23 (67.6%)	
Intestinal	10 (37.0%)	7 (20.6%)	
Mix	4 (14.8%)	4 (11.8%)	0.281
Bormann classification			
I-II	6 (22.2%)	1 (2.9%)	
III-IV	21 (77.8%)	33 (97.1%)	**0.019** ^∗^
Tumor size (max)			
>4 cm	16 (59.3%)	24 (70.6%)	
<4 cm	11 (40.7%)	10 (29.4%)	0.355
Pathological stage			
I-II	8 (29.6%)	7 (20.6%)	
III-IV	19 (70.4%)	27 (79.4%)	0.415
Tumor depth			
T1-T2	4 (14.8%)	5 (14.7%)	
T3-T4	23 (85.2%)	29 (85.3%)	0.990
Lymph node metastasis			
N0	10 (37.0%)	7 (20.6%)	
N1/N2/N3	17 (63.0%)	27 (79.4%)	0.155
Distant metastasis			
M0	21 (77.8%)	28 (82.4%)	
M1	6 (22.2%)	6 (17.6%)	0.655
Lymphatic vessel invasive (D2-40)			
Yes	6 (22.2%)	14 (41.2%)	
No	21 (77.8%)	20 (58.8%)	0.117
Vascular invasive (CD31)			
Yes	5 (18.5%)	10 (29.4%)	
No	22 (81.5%)	24 (70.6%)	0.326
Nerve invasive (S-100)			
Yes	17 (63.0%)	29 (85.3%)	
No	10 (37.0%)	5 (14.7%)	**0.044**∗
HER2 status			
0/1+/2+	22 (81.5%)	31 (91.2%)	
3+	5 (18.5%)	3 (8.8%)	0.265
PD-L1 status			
CPS ≥ 10	9 (33.3%)	15 (44.1%)	
CPS < 10	18 (66.7%)	19 (55.9%)	0.392

^∗^ *P* < 0.05 was considered significant.

## Data Availability

The raw reads were deposited into the NCBI SRA database (https://www.ncbi.nlm.nih.gov/sra/, accession number: SUB10789578 and Bioproject PRJNA812999).

## Supplementary Materials

Supplementary Materials Supplementary Figure 1: alpha diversity of the gastric mucosal fungal microbiota between the tumor and normal group. Alpha diversity indexes including the Chao1, Observed_species, Pielou_e, Shannon, Simpson, Faith's_pd, and Good's_coverage index were analyzed in the tumor and normal groups. Supplementary Figure 2: beta diversity of the gastric mucosal fungal microbiota between the tumor and normal groups. Beta diversity analysis, including PCoA and NMDS, was used to analyze the differences in the gastric microbiota community in samples based on the Bray-Curtis, Jaccard, Unweighted UniFrac, and Weighted UniFrac distance. Supplementary Figure 3: analysis of the components of the gastric fungal microbiota. (a, b) Principal component analysis (PCA) and orthogonal partial least squares discriminant analysis (OPLS-DA) revealed the main and separated clusters of the gastric fungal microbiota between the tumor and normal group. Supplementary Figure 4: Mann–Whitney *U* test was used to analyze the abundance of fungal microbiotas in the normal group. A total of 37 gastric mucosal fungal microbiotas that had higher abundance and were enriched significantly in the normal group compared to the tumor group were analyzed by the Mann–Whitney *U* test. Supplementary Figure 5: the role of *Solicoccozyma aeria* in the GC microenvironment. (a) Random forest classifier was used to calculate the importance of *Solicoccozyma aeria* in the grouping difference at the genus level; (b) the OTU abundance of *Solicoccozyma* at the species level; (c) a cluster heatmap showed the abundance of *Solicoccozyma aeria* in the primary tumor and corresponding paired normal tissues from 61 GC patients, and those patients with positive *Solicoccozyma aeria* expression in tumor were described; (d) the abundance of *Solicoccozyma aeria* in GC patients with stages I, II, III, and IV; (e) The ROC curves of *Solicoccozyma aeria* provided an area under the receiver operating characteristic curve (AUC) for the classification of the stage I and stage II-IV GC patients; (f) the abundance of *Solicoccozyma aeria* in GC patients with nerve invasion and nonnerve invasion; (g) The AUC value of *Solicoccozyma aeria* for the classification of the nerve invasive and nonnerve invasive GC patients; (h) the metabolic pathways were enriched in GC patients with *Solicoccozyma aeria-*positive expression in tumors. Supplementary Table 1: clinicopathological characteristics of *Solicoccozyma aeria* at species level in GC patients. ^∗^*P* < 0.05 was considered significant.
