# Supplementary material for: Dysbiosis of Gastric Mucosal Fungal Microbiota in the Gastric Cancer Microenvironment
Source: J Immunol Res. 2022 Mar 16;2022:6011632. doi: 10.1155/2022/6011632 (PMC8942701; doi:10.1155/2022/6011632)
Supplement: Supplementary Materials — Supplementary Figure 1: alpha diversity of the gastric mucosal fungal microbiota between the tumor and normal group. Alpha diversity indexes including the Chao1, Observed_species, Pielou_e, Shannon, Simpson, Faith's_pd, and Good's_coverage index were analyzed in the tumor and normal groups. Supplementary Figure 2: beta diversity of the gastric mucosal fungal microbiota between the tumor and normal groups. Beta diversity analysis, including PCoA and NMDS, was used to analyze the differences in the gastric microbiota community in samples based on the Bray-Curtis, Jaccard, Unweighted UniFrac, and Weighted UniFrac distance. Supplementary Figure 3: analysis of the components of the gastric fungal microbiota. (a, b) Principal component analysis (PCA) and orthogonal partial least squares discriminant analysis (OPLS-DA) revealed the main and separated clusters of the gastric fungal microbiota between the tumor and normal group. Supplementary Figure 4: Mann–Whitney U test was used to analyze the abundance of fungal microbiotas in the normal group. A total of 37 gastric mucosal fungal microbiotas that had higher abundance and were enriched significantly in the normal group compared to the tumor group were analyzed by the Mann–Whitney U test. Supplementary Figure 5: the role of Solicoccozyma aeria in the GC microenvironment. (a) Random forest classifier was used to calculate the importance of Solicoccozyma aeria in the grouping difference at the genus level; (b) the OTU abundance of Solicoccozyma at the species level; (c) a cluster heatmap showed the abundance of Solicoccozyma aeria in the primary tumor and corresponding paired normal tissues from 61 GC patients, and those patients with positive Solicoccozyma aeria expression in tumor were described; (d) the abundance of Solicoccozyma aeria in GC patients with stages I, II, III, and IV; (e) The ROC curves of Solicoccozyma aeria provided an area under the receiver operating characteristic curve (AUC) for the classification of t [file 6011632.f1.docx]

Supplementary Material

**Supplementary Figure.1 Figure Legend**

**Alpha-diversity of the gastric mucosal fungal microbiota between the tumor and normal group.**

Alpha-diversity indexes included the Chao1, Observed_species, Pielou_e, Shannon, Simpson, Faith’s_pd and Good’s_coverage index were analyzed in the tumor and normal group.


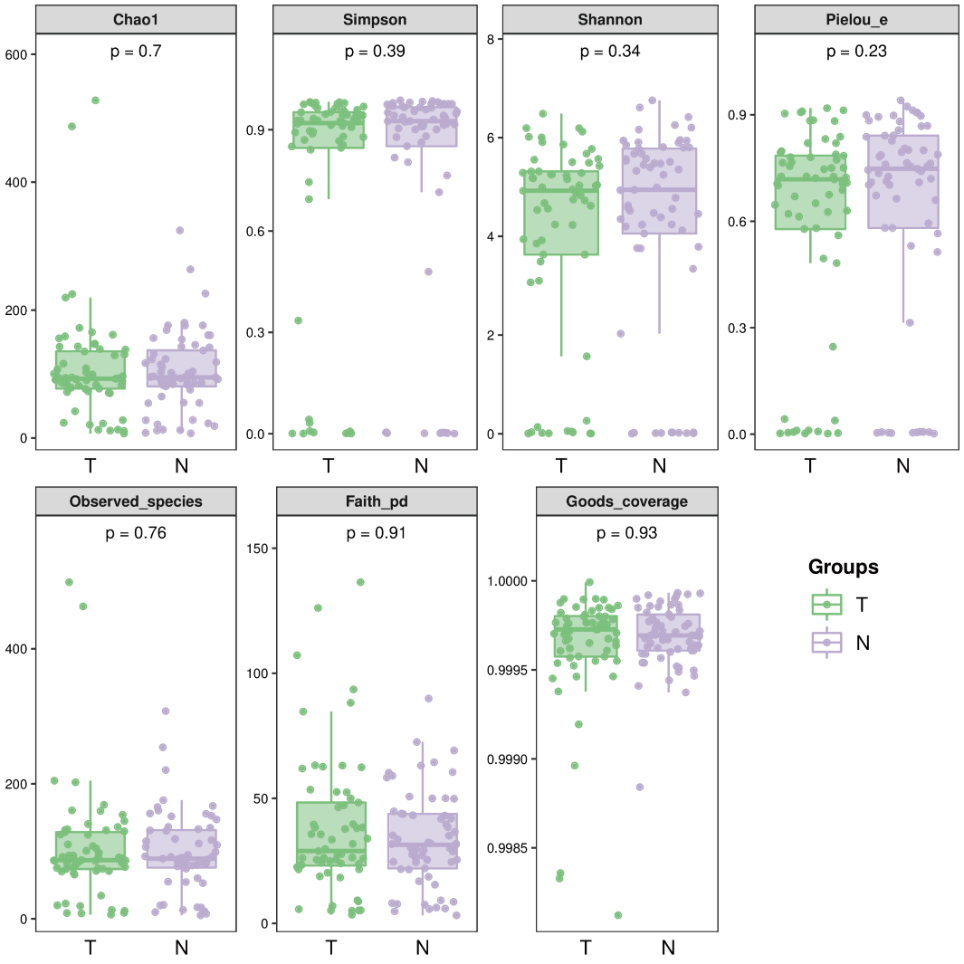


**Supplementary Figure.2 Figure Legend**

**Beta-diversity of the gastric mucosal fungal microbiota between the tumor and normal groups.**

Beta-diversity analysis, including PCoA and NMDS, was used to analyze the differences in the gastric microbiota community in samples based on the Bray-Curtis, Jaccard, Unweighted UniFrac and Weighted UniFrac distance.


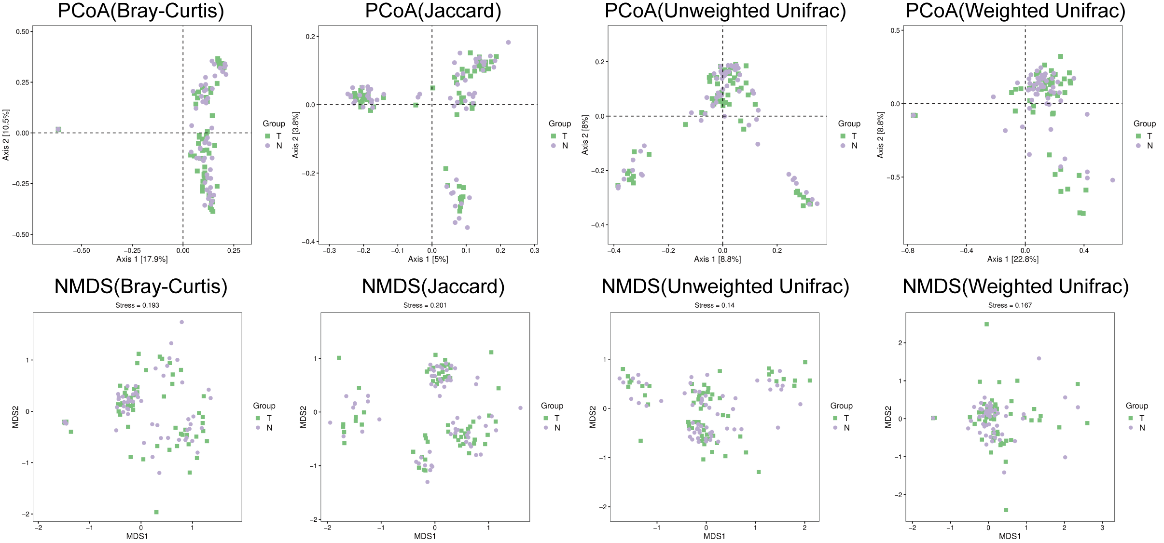


**Supplementary Figure.3 Figure Legend**

**Analysis of the components of the gastric fungal microbiota**


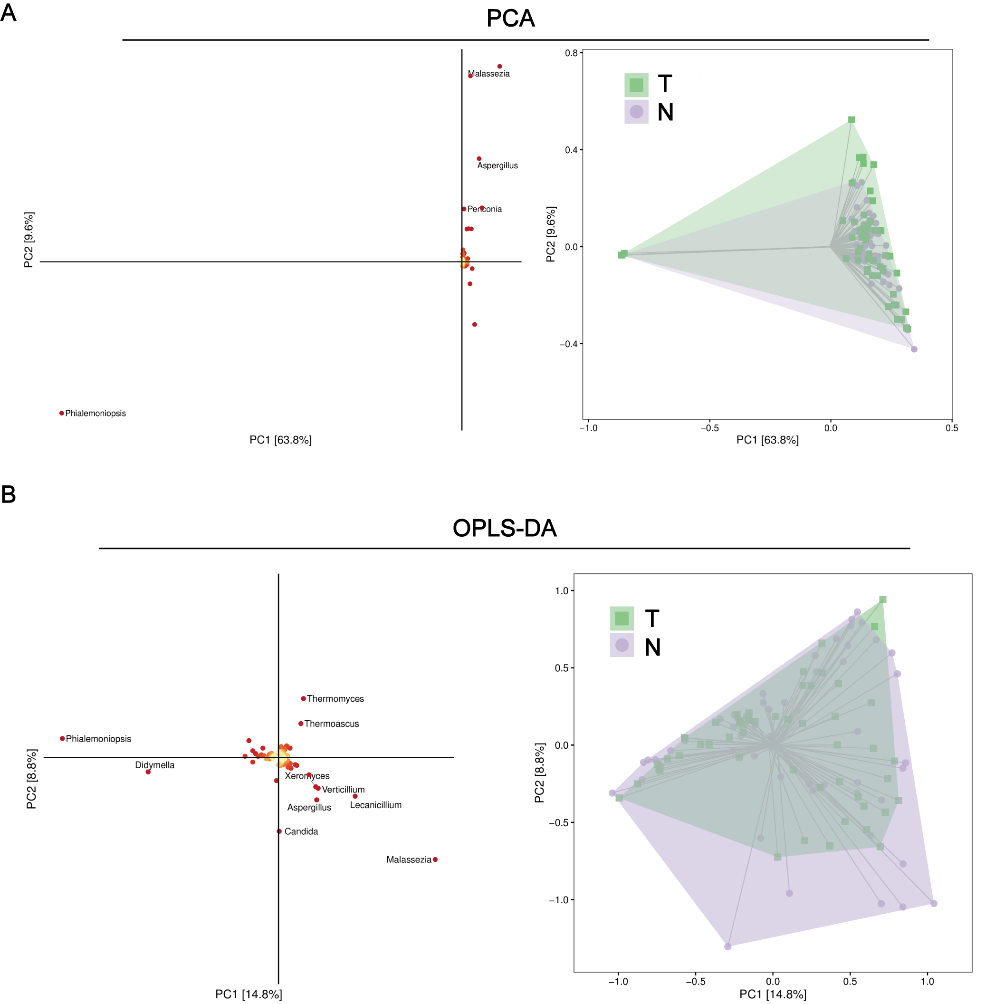
(A&B) Principal component analysis (PCA) and orthogonal partial least squares discriminant analysis (OPLS-DA) revealed the main and separated clusters of the gastric fungal microbiota between the tumor and normal group.

.

**Supplementary Figure.4 Figure Legend**

**Mann–Whitney U test was used to analyze the abundance of fungal microbiotas in the normal group.**

A total of 37 gastric mucosal fungal microbiotas that had higher abundance and were enriched significantly in the normal group compared to the tumor group, were analyzed by the Mann–Whitney U test.


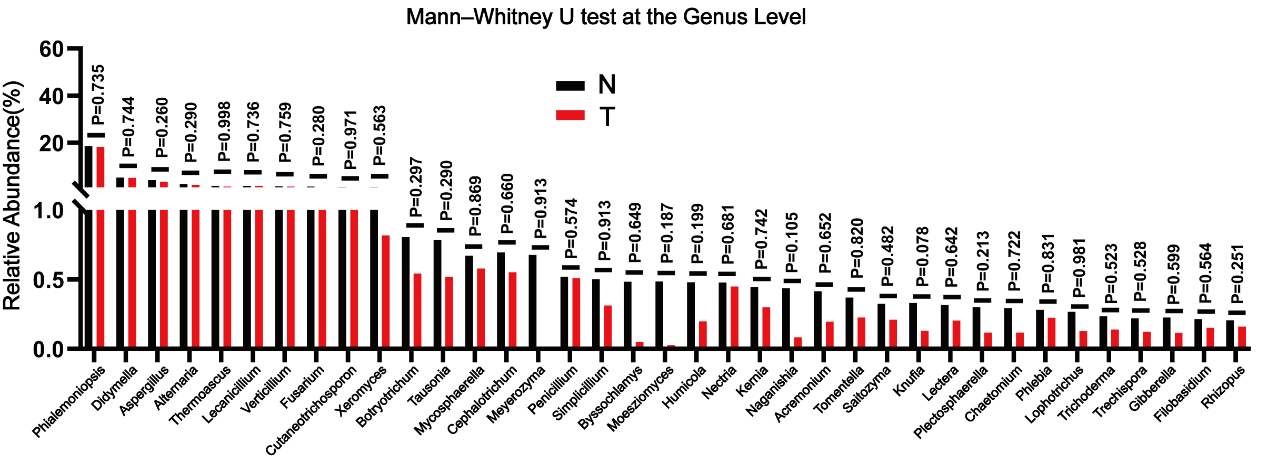


**Supplementary Figure.5 Figure Legend**

**The role of *Solicoccozyma aeria* in the GC microenvironment**

(A) Random forest classifier was used to calculate the importance of *Solicoccozyma aeria* in the grouping difference at the genus level; (B) The OTU abundance of *Solicoccozyma* at the species level; (C) A cluster heatmap showed the abundance of *Solicoccozyma aeria* in the primary tumor and corresponding paired normal tissues from 61 GC patients, and those patients with positive *Solicoccozyma aeria* expression in tumor were described; (D) The abundance of *Solicoccozyma aeria* in GC patients with stage I, II, III and IV; (E) The ROC curves of *Solicoccozyma aeria* provided an area under the receiver operating characteristic curve (AUC) for the classification of the stage I and stage II-IV GC patients; (F) The abundance of *Solicoccozyma aeria* in GC patients with nerve invasion and non-nerve invasion; (G) The AUC value of *Solicoccozyma aeria* for the classification of the nerve invasive and non-nerve invasive GC patients; (H) The metabolic pathways were enriched in GC patients with *Solicoccozyma aeria-*positive expression in tumors.


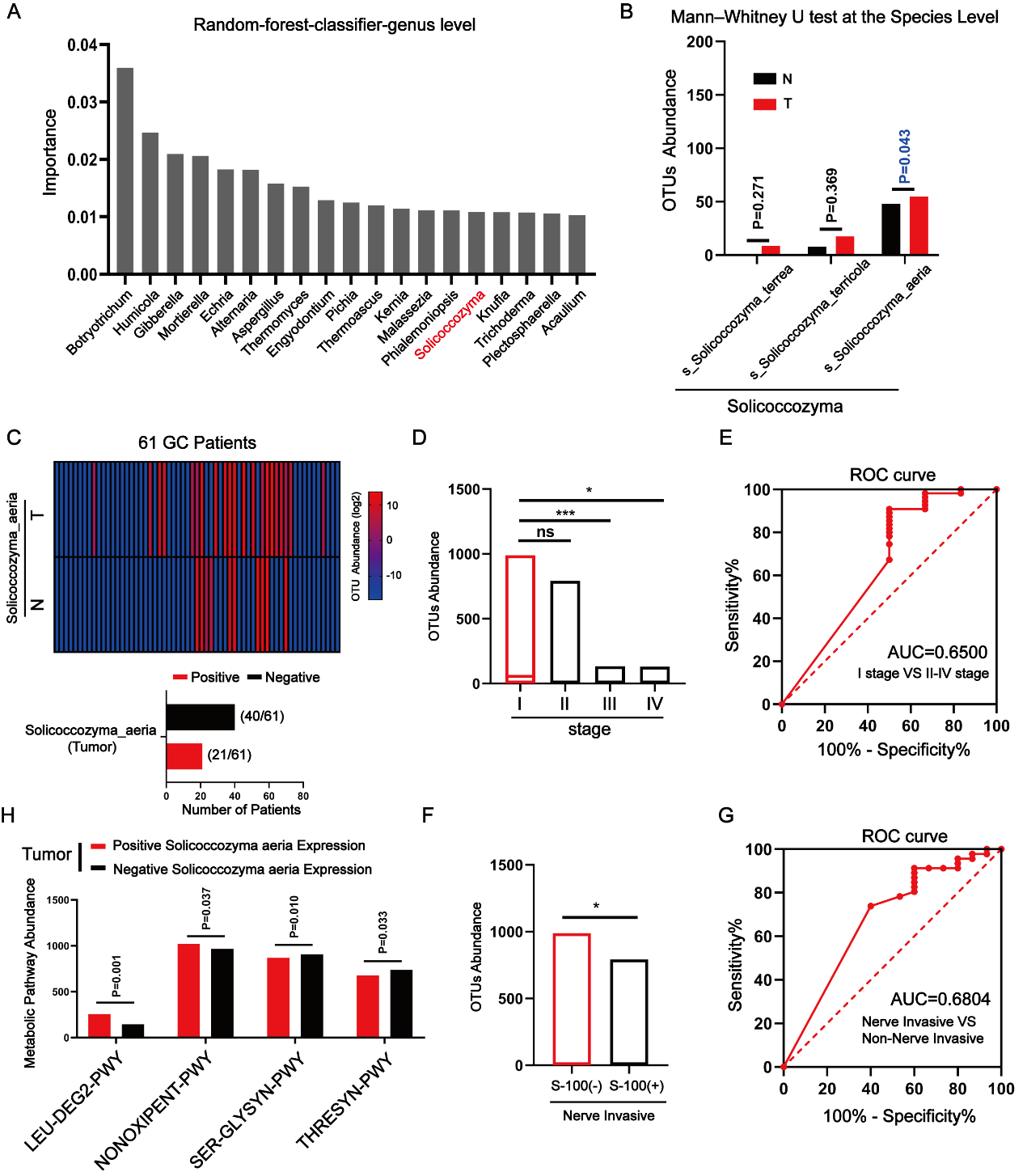


**Supplymentary.Table.1** Clinicopathological characteristics of *Solicoccozyma aeria* at species level in GC patients.

| Characteristics |  | | *Solicoccozyma aeria* expression | | |  |
| --- | --- | --- | --- | --- | --- | --- |
|  |  | Positive (n=21) | | | Negative (n=40) | P-value |
| Age | ≥ 60 | | 11 (52.4%) | 16 (40%) | |  |
|  | <60 | | 10 (47.6%) | 24 (60%) | | 0.355 |
| Gender | Male | | 13 (61.9%) | 30 (75.0%) | |  |
|  | Female | | 8 (38.1%) | 10 (25.0%) | | 0.287 |
| Body Mass Index | <18 | | 1 (4.7%) | 2 (5%) | |  |
|  | 18-24 | | 14 (66.7%) | 27 (67.5%) | |  |
|  | > 24 | | 6 (28.6%) | 11 (27.5%) | | 0.996 |
| Tumor Location | Upper | | 7 (33.3%) | 12 (30%) | |  |
|  | Middle/Lower | | 14 (66.7%) | 28 (70%) | | 0.789 |
| Tumor Differentiation | High | | 2 (9.5%) | 2 (5%) | |  |
|  | Moderately/Poor | | 19 (90.5%) | 38 (95%) | | 0.498 |
| Lauren Classification | Diffuse | | 10 (47.6%) | 26 (65%) | |  |
|  | Intestinal | | 9 (42.9%) | 8 (20%) | |  |
|  | Mix | | 2 (9.5%) | 6 (15%) | | 0.165 |
| Bormann Classification | I-II | | 5 (23.8%) | 2 (5%) | |  |
|  | III-IV | | 16 (76.2%) | 38 (95%) | | **0.029*** |
| Tumor Size (Max) | >4cm | | 12 (57.1%) | 28 (70%) | |  |
|  | <4cm | | 9 (42.9%) | 12 (30%) | | 0.315 |
| Pathological Stage | I-II | | 7 (33.3%) | 8 (20%) | |  |
|  | III-IV | | 14 (66.7%) | 32 (80%) | | 0.251 |
| Tumor Depth | T1-T2 | | 3 (14.3%) | 6 (15%) | |  |
|  | T3-T4 | | 18 (85.7%) | 34 (85%) | | 0.940 |
| Lymph Node Metastasis | N0 | | 9 (42.9%) | 8 (20%) | |  |
|  | N1/N2/N3 | | 12 (57.1%) | 32 (80%) | | 0.059 |
| Distant Metastasis | M0 | | 17 (81.0%) | 32 (80%) | |  |
|  | M1 | | 4 (19.0%) | 8 (20%) | | 0.929 |
| Lymphatic Vessel Invasive (D2-40) | Yes | | 3 (14.3%) | 17 (42.5%) | |  |
|  | No | | 18 (85.7%) | 23 (57.5%) | | **0.026*** |
| Vascular Invasive (CD31) | Yes | | 3 (14.3%) | 12 (30%) | |  |
|  | No | | 18 (85.7%) | 28 (70%) | | 0.176 |
| Nerve Invasive (S-100) | Yes | | 12 (57.1%) | 34 (85%) | |  |
|  | No | | 9 (42.9%) | 6 (15%) | | **0.016*** |
| HER2 Status | 0/1+/2+ | | 16 (76.2%) | 37 (92.5%) | |  |
|  | 3+ | | 5 (23.8%) | 3 (7.5%) | | 0.073 |
| PD-L1 Status | CPS≥10 | | 7 (33.3%) | 17 (42.5%) | |  |
|  | CPS<10 | | 14 (66.7%) | 23 (57.5%) | | 0.486 |

*P<0.05 was considered significant.
